# Functions of silicon in plant drought stress responses

**DOI:** 10.1038/s41438-021-00681-1

**Published:** 2021-12-01

**Authors:** Min Wang, Ruirui Wang, Luis Alejandro Jose Mur, Jianyun Ruan, Qirong Shen, Shiwei Guo

**Affiliations:** 1 Jiangsu Provincial Key Lab of Solid Organic Waste Utilization, Jiangsu Collaborative Innovation Center of Solid Organic Wastes, The Key Laboratory of Plant Immunity, Nanjing Agricultural University, Nanjing, 210095 Jiangsu China; 2 Institute of Biological, Environmental and Rural Sciences, Aberystwyth University, Aberystwyth, SY23 3DA UK; 3 Key Laboratory of Tea Plant Biology and Resources Utilization (Ministry of Agriculture), Tea Research Institute, Chinese Academy of Agricultural Sciences, Hangzhou, 310008 Zhejiang China

**Keywords:** Drought, Plant physiology

## Abstract

Silicon (Si), the second most abundant element in Earth’s crust, exerts beneficial effects on the growth and productivity of a variety of plant species under various environmental conditions. However, the benefits of Si and its importance to plants are controversial due to differences among the species, genotypes, and the environmental conditions. Although Si has been widely reported to alleviate plant drought stress in both the Si-accumulating and nonaccumulating plants, the underlying mechanisms through which Si improves plant water status and maintains water balance remain unclear. The aim of this review is to summarize the morphoanatomical, physiological, biochemical, and molecular processes that are involved in plant water status that are regulated by Si in response to drought stress, especially the integrated modulation of Si-triggered drought stress responses in Si accumulators and intermediate- and excluder-type plants. The key mechanisms influencing the ability of Si to mitigate the effects of drought stress include enhancing water uptake and transport, regulating stomatal behavior and transpirational water loss, accumulating solutes and osmoregulatory substances, and inducing plant defense- associated with signaling events, consequently maintaining whole-plant water balance. This study evaluates the ability of Si to maintain water balance under drought stress conditions and suggests future research that is needed to implement the use of Si in agriculture. Considering the complex relationships between Si and different plant species, genotypes, and the environment, detailed studies are needed to understand the interactions between Si and plant responses under stress conditions.

## Introduction

Silicon (Si) is the second most abundant mineral element present in the soil, and silicon dioxide composes approximately 50–70% of the soil mass^1–4^. Si has various ecological functions, with complex roles in plant processes and in mediating interactions with the environment and other organisms^5–7^. Si accumulation varies greatly among plant species, ranging from 0.1 to 10% dry weight. Based on the Si content in tissues, plants can be classified as accumulator (e.g., rice, wheat, maize, and sorghum), intermediate (e.g., cucumber, bitter gourd, and melon), or excluder (e.g., tomato, potato, canola, and lentil) types^8,9^. The differences are attributed to the different modes of Si uptake (active, passive, and rejective)^10,11^. In addition, these differences are largely due to the abilities of the roots of various plant species to absorb Si^4^, which is related to Si transporter expression and function. Below a pH of nine, Si is generally taken up by plant roots in the form of silicic acid [Si(OH)_4_], an uncharged monomeric molecule^4^ that is dependent primarily on a specific Si influx transporter (Lsi1) and a specific efflux transporter (Lsi2). Another influx transporter, Lsi6, regulates the unloading of Si from the xylem to leaf tissues and further facilitates root-to-shoot translocation^4,12,13^. In addition to Si taken up by roots, Si fertilizer can also be efficiently supplied to leaves to increase plant dry matter production^14–17^ and is absorbed mainly via cuticular pathways, stomata, and trichomes^18^. Foliar application of Si-containing solutions is a viable alternative Si fertilization method to increase Si accumulation, especially for intermediate and Si nonaccumulator plants^15,16,19,20^.

During their growth and development, plants are subjected to various environmental stresses. Si has been widely reported to enhance plant tolerance to various abiotic and biotic stresses, such as drought, salt, freezing, nutrient imbalance, radiation damage, metal toxicity, pests, and pathogens^5,21–26^. Drought, a recurring phenomenon with major impacts on both humans and natural ecosystems, is the most widespread climatic extreme that hinders primarily crop growth and productivity^27^. In this context, the alleviating effects of Si on drought stress has been observed in a wide variety of crop plants species, including both monocots (e.g., rice, wheat, maize, and sorghum) and dicots (e.g., tomato, cucumber, sunflower, soybean, cotton, mango, and canola)^28–38^. Interestingly, Si has been shown to counteract the effects of drought stress in plant species that have a weakly ability to accumulate Si (Si excluders), such as tomato and canola. Additionally, wheat landraces that were high Si accumulators had higher levels of shoot Si compared to low accumulators, but no differences in growth or stress tolerance were observed underwater stress^39^. This suggests that the effects of Si are not proportional to its accumulation in plants and that a low amount of Si accumulation does not equate to poor function^40^. The role of Si in low Si-accumulating plants is attributed mainly to the biochemical function of Si, while mechanical/physical barriers induced by Si deposition in high Si-accumulating plants are important for the stress response^7,32,41^. For example, Si also was shown to induce resistance to bacterial wilt disease caused by *Ralstonia solanacearum* in Si-nonaccumulating tomato plants, which was mediated mainly via signaling pathways, such as those involving ethylene (ET), jasmonic acid (JA), and/or reactive oxygen species (ROS)^42^.

Although Si is not considered an essential element for plants, it is well known to be beneficial for plant growth and development, especially under stress conditions^2,5,43^. Si stimulates seed germination in wheat, maize, lentil, and tomato under drought stress^41,44–46^, the effects of which are attributed to the increased antioxidant defense and decreased oxidative stress induced by Si^41,47^. During plant growth, Si has been found to increase plant biomass and grain yields of several crop species under drought stress^29,35,48,49^, which is attributed to increases in total root length, surface area, and volume as well as increases in plant height, dry matter, panicle length, and tiller number^28,48,50^. Another important feature due to the possible role of Si is reducing spikelet sterility and subsequently increasing the grain yields of rice supplied with Si^28,48,50^.

**Fig. 1:**
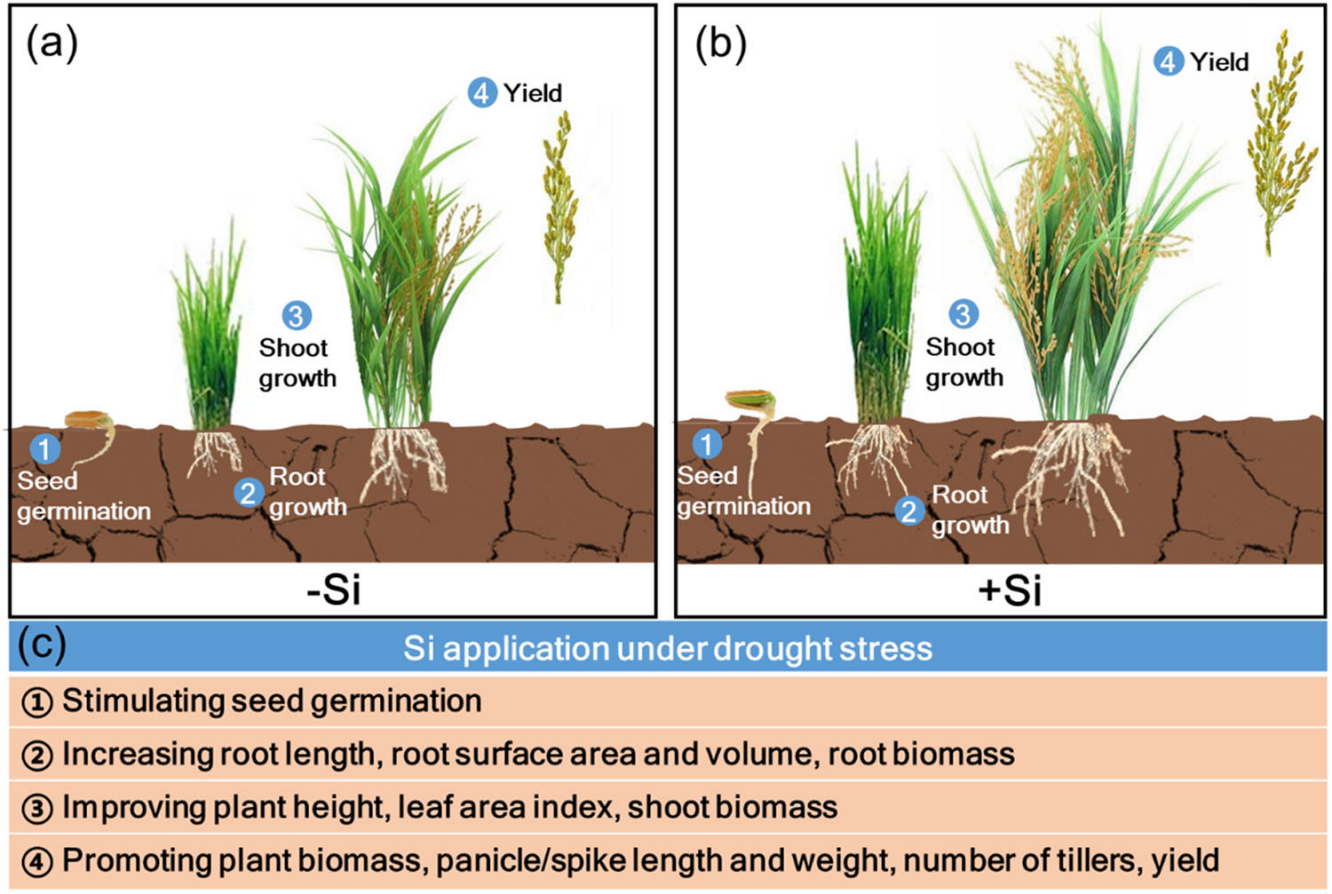
Beneficial effects of silicon (Si) on the growth and development of plants under drought stress. **a** Plant growth and yield production in the absence of Si application (-Si). Seed germination, root growth, shoot growth, and crop yields are suppressed by drought without Si application. **b** Plant growth and yield production in the presence of Si application (+Si). **c** The beneficial effects of Si under drought stress include stimulating seed germination (1) and increasing both root (2) and shoot growth (3), thus increasing plant biomass and yield (4) under drought stress

Given the obvious benefits of Si on drought tolerance (Fig. 1), it may be expected that its process has been extensively characterized. However, the detailed mechanisms remain unknown and appear to vary according to genotype and environment. In this review, the morphoanatomical, physiological, biochemical, and molecular processes by which Si alleviates plant drought stress, especially the potential functions of Si in the accumulator, intermediate, and excluder plants, are summarized. This study provides an overview of the currently available information on Si-mediated root water uptake, leaf water loss, and plant defense responses under drought stress.

## Si increases root water uptake under drought stress

### Improving root/shoot ratios

Increasing root water uptake by regulating the root surface and anatomy is important for plant stress tolerance^51^. Si is essential for root development and water uptake under drought stress conditions^49,52^. It was suggested that Si application regulates polyamine (PA) and 1-aminocyclopropane-1-carboxylic acid (ACC) levels under drought stress conditions to increase root growth and the root/shoot ratio^53^, thus improving root water uptake^28,32,53–56^. Such Si-mediated changes in root development also increase root endodermal silicification and suberization^54,55^, therefore enhancing the capability of water retention to overcome the effects of drought stress. Root endodermal development involves three main stages: Casparian band formation, deposition of suberin lamellae, and thickening of cell walls. Si has been shown to promote Casparian band development by crosslinking phenols with the cell wall or by inducing precipitation of phenols^56^. Endodermal silicification associated with cell walls in the roots is arranged in a specific pattern that initiates in endodermal cells adjacent to the phloem, continues to the xylem poles, and is ultimately observed in so-called passage cells^57^. However, in a study of sorghum, endodermal silicification-induced drought resistance was not driven through an improved root water retention capability, and root silicification might help overcome drought stress by decreasing root growth inhibition caused by desiccation^58^.

In contrast, several researchers have reported no effects of Si on the root/shoot ratio but have reported increases in both the root and shoot dry weight under stress conditions^32,52,59^, and these authors suggested that Si was effective at improving plant resistance to osmotic stress and that root hydraulic conductance is important for Si-promoted root water uptake^31^. Thus, Si-enhanced water uptake under drought stress conditions could be specific to plant species, genotype, or even environmental conditions. In the following section, the functions of Si in water uptake and transport are discussed.

### Promoting the root osmotic driving force

Osmotic adjustment and accumulation of compatible cellular solutes are considered plant physiological processes that occur in response to drought stress^60,61^. These adjustments are attributed mainly to turgor maintenance and the protection of specific cellular functions by the accumulation of compatible organic solutes such as amino acids, soluble sugars, and minerals^62,63^, resulting in a favorable osmotic gradient between the plant roots and the growth medium to facilitate water uptake^51,64,65^.

An increasing number of studies have indicated that applying Si promotes osmolyte accumulation in many plant species, especially Si accumulators, such as rice, wheat, maize, and sorghum, under drought stress^28–30,66^, thus improving the osmotic driving force for water uptake^66^. In line with this point, Si has been reported to regulate the activities of enzymes involved in carbohydrate metabolism and affect the lignification of cell walls, consequently regulating assimilate synthesis and transport efficiency^28–30,38,66–68^. Other osmotic responses are exhibited by cucumber and wheat plants, which show increased protein content when exposed to salt and drought stress together with Si^29,69^, and also in chickpea and sunflower plants, in which proline accumulation is induced by Si under drought stress^34,70^. The accumulation of these osmolytes involves not only osmotic adjustment but also detoxification of ROS, maintenance of membrane integrity, and stabilization of proteins/enzymes, which contribute to drought tolerance. However, another study in tomato (a Si excluder) showed that osmotic events were not affected by Si under drought^32^, suggesting that the Si-mediated increase in root water uptake was not due to an increase in the osmotic driving force under drought stress but rather was due to an improved root hydraulic conductance. In addition, Si application alleviated drought stress by decreasing the content of osmolytes in lentil and potato plant species (Si excluders)^46,67^, suggesting that the role of the osmotic driving force in Si-mediated improvement of water uptake differs between the Si accumulators and excluders. Therefore, the osmotic driving force was not the only important response, and the role of the osmotic driving force in the Si-mediated enhancement of water uptake does not appear to be deployed in all situations.

The abovementioned studies implied that Si application increased plant drought tolerance by regulating osmotic adjustments based on organic solute accumulation. However, since little is known about the mechanisms of Si-mediated osmotic adjustment in plants, the relationship between the Si application and plant-compatible solute metabolism needs future investigation, especially the difference between the Si accumulators and excluders.

### Increasing root hydraulic conductance

The root water uptake capacity is largely determined by hydraulic conductance^71^, and Si application has been reported to improve root hydraulic conductance in Si accumulators, intermediates, and excluders plants underwater and salt stress^31,32,59,66,72–75^. Root hydraulic conductance can be inhibited by high exogenous hydrogen peroxide (H_2_O_2_) levels, which are correlated with membrane electrolyte leakage and ROS levels^76^. H_2_O_2_ is involved in the formation of suberin lamellae, which form a hydrophobic barrier in the endodermis and exodermis of roots^77^. Under stress conditions, Si application reduces H_2_O_2_ production and suberin lamella formation and further induces increased water permeability^32^. In tomato plants under drought stress, root plasma membrane integrity was improved in response to Si application, and negative correlations were found between root hydraulic conductance and the levels of both the ROS and lipid peroxidation products^32^. The Si-mediated alleviation of ROS production under drought stress corresponded with an increase in antioxidant defenses, mainly attributed to the improved activity of catalase (CAT) and superoxide dismutase (SOD), as well as contents of ascorbic acid (AsA) and reduced glutathione (GSH)^32^. Therefore, the enhanced root hydraulic conductance and water uptake in response to Si could arise from a reduction in membrane oxidative damage^32^. In addition, Si-mediated transcriptional upregulation of root aquaporin genes contributed to increased hydraulic conductance and water uptake under drought stress^31^. It has been reported that oxidative damage causes plasma membrane dysfunction; thus, the overproduction of ROS under drought stress may negatively regulate the activities of plasma membrane aquaporins^32^. The role of aquaporins in root water uptake regulated by Si under drought stress is discussed in the following sections.

Overall, the modification of root growth and hydraulic conductance in response to Si application enhances root water uptake under drought stress conditions. A Si-mediated reduction in membrane oxidative damage via increased antioxidant defense may contribute to enhanced root hydraulic conductance. Further studies are needed to investigate how Si regulates root development under drought stress conditions. Specifically, the complex interactions between membrane oxidative damage and ROS accumulation in root hydraulic conductance need to be determined.

### Regulation of aquaporins (AQPs)

Aquaporins belong to the major intrinsic protein (MIP) family and regulate the transport of water and small solutes across membranes^78–82^, contributing to root water uptake, especially under drought stress conditions^31,71,83,84^. Water moves within the roots both radially from the root surface into xylem vessels and axially along the xylem^85^, while aquaporins mainly function in radial water movement in both the water uptake and transport. There are three main pathways for water flow in radial movement: the apoplastic, symplastic, and transcellular pathways^85^. The symplastic and transcellular pathways are collectively referred to as the cell-to-cell pathway^86^, which is mainly dependent on aquaporins^87^.

In the presence of Si, there is a dual role played by aquaporins under drought stress. On the one hand, Lsi1, a Si-permeable channel, belongs to a NOD26-like intrinsic protein (NIP) subfamily of aquaporins, which are involved in Si transport^12,88,89^. As Si accumulation in plants requires the dual action of both the influx and efflux transporters, the Si transporter Lsi1 has evolved a unique selective amino acid filter, which is one of the required features to regulate the influx of Si and the indispensable key for plants to absorb Si^12,90^. On the other hand, Si induces the expression of aquaporin genes to increase root water uptake^73,91^; for example, in sorghum plants, Si application markedly enhances aquaporin activity via the upregulation of the *SbPIP1;6*, *SbPIP2;2*, and *SbPIP2;6* genes, consequently increasing root water uptake by enhancing root hydraulic conductance under drought stress^31,91,92^. However, inconsistent results were observed in a Si excluder (tomato), and the expression of the *SlPIP1;3*, *SlPIP1;5*, and *SlPIP2;6* genes was not significantly affected after Si application under drought stress^32^, suggesting that Si did not improve root water uptake by upregulating aquaporin genes in tomato roots but instead did so by increasing root hydraulic conductance (as mentioned above).

Therefore, the ability of Si to alleviate drought stress is mainly attributed to its direct effect through regulating the activity of aquaporins and gene expression, as well as its indirect effect through increasing root hydraulic conductance (personal communication with Rony Wallach, Hebrew University of Jerusalem). However, the molecular mechanism of Si-mediated alleviation of drought stress is poorly understood, and the genes related to water uptake and osmotic adjustment regulated by Si need to be determined. Further studies should focus on the underlying interactions between the Si and processes related to water relations (water uptake, transport, and loss) under stress conditions.

### Enhancing mineral nutrient uptake and maintaining nutrient balance

Mineral nutrient uptake and homeostasis can be disrupted by environmental stimuli, especially drought stresses^34,48^. It has been reported that the uptake of nitrogen (N), phosphate (P), potassium (K), calcium (Ca), magnesium (Mg), iron (Fe), copper (Cu), and manganese (Mn) increases in response to Si application under drought stress^30,34,48,93^, which not only enhances plant growth but also improves plant resistance and/or tolerance. For example, K and Ca contents were considerably increased in maize in response to Si application under drought stress^30^, in which K benefits plant growth, osmotic adjustment, and drought tolerance^94^, and Ca is critical for achieving better survival with improved plant growth^95^, maintaining the integrity of plant membranes and regulating ion permeability and selectivity^96^.

The possible mechanisms for Si-induced mineral nutrient uptake include (i) increasing water uptake and transpirational driving forces^31,92^, thus enhancing mineral nutrient movement from soil into roots; (ii) enhancing ion mobilization in roots (e.g., Si alleviates Fe deficiency in cucumber by increasing the apoplastic Fe pool in the roots and enhancing Fe mobilization in the roots due to Si-mediated biosynthesis of Fe-chelating compounds)^97^; (iii) stimulating membrane H ^+ ^-ATPase activity driving mineral nutrient uptake (e.g., Si increased K ^+ ^uptake in barley under osmotic stress by activating H ^+ ^-ATPase in the membranes)^98^; (iv) regulating ion transporter genes (e.g., Si modulates the activities and gene expression of enzymes involved in Fe acquisition in cucumber)^97^, while Si also regulates genes involved in Mn and Cd uptake and translocation in rice^99,100^; and (v) enhancing the translocation of metabolites that contribute to root/shoot ion transport (e.g., Si increases micronutrient transport and distribution by increasing the content of long-distance molecules, such as citrate)^101^. In brief, the uptake of essential nutrients in response to Si application under drought stress maintains the nutrient balance, thereby increasing water uptake and improving plant resistance to environmental stress.

**Fig. 2:**
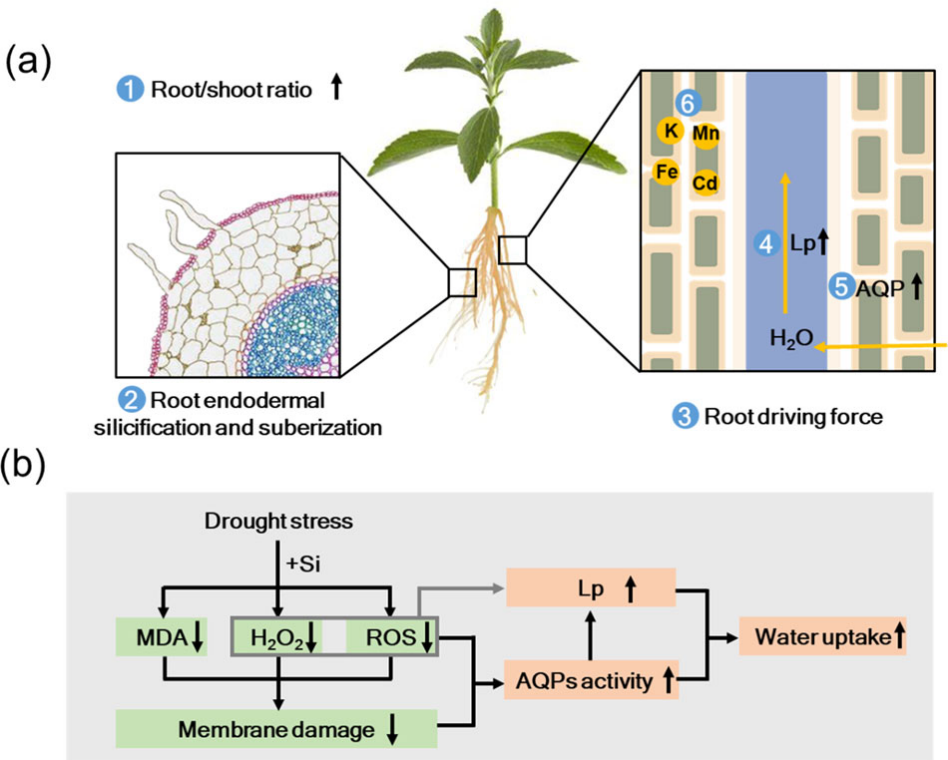
Water uptake increases in response to Si application under drought stress conditions. **a** Silicon (Si) application acts via the following mechanisms: (1) increasing the root/shoot ratio; (2) inducing root endodermal silicification and suberization; (3) enhancing the root driving force; (4) improving root hydraulic conductance (Lp); (5) increasing aquaporin (AQP) activity; and (6) maintaining nutrient balance. **b** Root hydraulic conductance and aquaporins are regulated by Si under drought stress. Si application improves root Lp by inhibiting reactive oxygen species (ROS) and hydrogen peroxide (H_2_O_2_) production and increases AQP activity by reducing ROS production and membrane damage, thus improving water uptake

In summary, the beneficial effects of Si on water uptake may be attributed to the improvement in root growth, driving force, root hydraulic conductance, aquaporin activity, and gene expression, as well as the maintenance of nutrient balance (Fig. 2). The interactions between the Si and other essential nutrients under drought stress are worthy of further study to explore the role of Si in root water uptake.

## Si regulates leaf water loss under drought stress

Numerous researchers have shown that Si application regulates gas exchange, which in turn contributes to drought tolerance, in species such as maize^93,102^, soybean^103^, cucumber^104^, and alfalfa^105^; this ultimately resulted in increased water-use efficiency (WUE) and the alleviation of drought stress^93^. In previous studies, Si-induced reduction in transpiration was considered to be the result of physical blockade of cuticular transpiration via cuticle layer thickening from silica deposits^106–108^, which contributes to the maintenance of leaf water potential underwater-deficient conditions^54^. For example, wheat leaves are thicker after Si application under drought, thus reducing transpirational water loss^109,110^. However, in maize plants, it was suggested that the lower transpiration of Si-supplied plants was primarily due to stomatal pores rather than the cuticular layer^93,102^, mainly attributed to the loss of guard cell turgor and changes in the physical and mechanical properties of the cell walls^111–113^.

In contrast to the abovementioned observations, some reports have suggested that Si application increased the leaf transpiration rate in rice, tomato, pepper, mangrove, and sorghum under drought stress^31,32,48,114,115^. This increased transpiration was attributed to an improvement in leaf water status via increased water uptake, enhanced leaf xylem sap flow, and increased leaf water potential resulting from a larger leaf area^110^. Such results were also consistent with those of Zhang et al.^116^, who suggested that Si-improved plant growth may be attributed to increased gas exchange parameters, e.g., transpiration and stomatal conductance. However, it has also been reported that Si has no effect on the transpiration rates of cucumber and rose plants under drought stress conditions^117,118^, implying that Si-regulated transpiration is dynamic and depends on root water status, environmental conditions, plant species, and genotype.

**Fig. 3:**
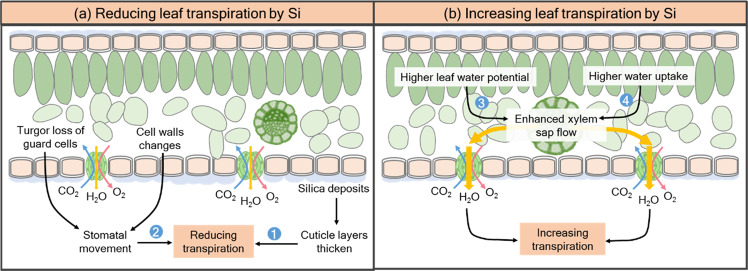
Si influences leaf transpiration under drought stress. **a** Leaf transpiration can be reduced by Si application under drought stress via (1) physical blockade of cuticular transpiration via cuticle layer thickening caused by silica deposits and via (2) regulation of stomatal movement by turgor loss of guard cells and by changes in the physical and mechanical properties of cell walls. **b** In contrast, Si application increased the leaf water potential (3) and water uptake (4), thus enhancing leaf xylem sap flow and transpiration under drought stress conditions. In addition, Si has also been reported to have no effect on leaf transpiration in some cases

The role of Si in alleviating drought stress by regulating transpiration is summarized in Fig. 3. When root water uptake was limited, this model suggested that the Si supply decreased leaf transpiration to reduce water loss by physically blocking cuticular transpiration or stomatal movement. In contrast, Si increased leaf xylem sap flow and transpiration rates under drought stress, corresponding to increased photosynthesis rates. The differential impact of Si on transpiration rates may be related to the degree of stress. Under mild stress conditions, Si could increase root water uptake, corresponding to increased transpiration rates, and consequently increase plant growth under drought stress. When root water uptake is limited under heavy stress, plant leaves close their stomata to reduce water loss, which occurs most likely through a systemic signaling event(s). More broadly, leaf transpiration exerts feedback effects on root water transport models^71^. With high leaf transpiration rates, the transpiration force driving water across the roots mainly depends on the hydrostatic pressure difference between the root medium and xylem, which allows both the apoplastic and cell-to-cell pathways to be used. When transpiration is reduced, only a cell-to-cell process is available, which has high hydraulic resistance^71^. Nonetheless, detailed studies are still needed to understand the mechanisms of Si in whole-plant water relations and to consider the complex relationship between Si supply and transpiration in plants under drought.

## Si invokes plant defense responses under drought stress

### Modification of signaling pathways

To alleviate environmental stress, plants have developed a complex signal transduction network. Si application has been reported to increase plant tolerance by regulating endogenous plant phytohormone balance and associated signaling events, including those involving abscisic acid (ABA), JA, salicylic acid (SA), and ET^22,53,119–121^. For example, Si addition enhanced the drought tolerance of sorghum, at least in part, by regulating the synthesis of PAs, as well as ACC, the precursor of ET^53^. Furthermore, Si decreased JA contents in soybean under drought^122^, which suggested Si inhibited an early signaling event required for JA production. ABA, a stress-responsive hormone, plays an essential role in stomatal closure when plants are exposed to various environmental stresses^123^. In barley plants, Si application did not affect ABA levels in the leaves under normal conditions but decreased ABA homeostasis via transcriptional regulation of ABA biosynthesis and degradation pathways, thus improving stress tolerance^124^.

Several studies have proposed that Si mediates the modulation of multiple genes involved in stress-responsive pathways via the JA, ABA, and phenylpropanoid pathways^125–128^. In rice, Si regulates the transcription factors *OsNAC5* and *OsDREB2A*, which trigger the expression of stress-responsive genes that impart tolerance to osmotic stress via ABA-dependent and ABA-independent pathways, respectively^129,130^. The Si-dependent upregulation of transcription factors could interact with *cis*-elements located in the promoter regions of genes involved in the stress response and trigger tolerance to abiotic and biotic stresses^126^. Given the current knowledge of these phytohormone signaling pathways, the means through which Si impacts particular components and affects crosstalk between signals under stress conditions must be urgently addressed.

### Activation of the antioxidant system

The balance between ROS and antioxidants is disrupted by environmental stresses, resulting in oxidative damage to membrane lipids^131,132^. The antioxidative processes that reduce ROS in plant cells include both the enzymes [e.g., SOD, CAT, peroxidase (POD) and ascorbate peroxidase (APX)] and nonenzymatic compounds [e.g., AsA, GSH, tocopherols, and carotenoids]^29,41^. ROS accumulation under drought stress is inversely correlated with the activities of plasma membrane aquaporins^76^. Indeed, aquaporin phosphorylation status and intracellular trafficking are regulated by ROS-dependent signaling mechanisms^133^. Therefore, the regulation of water movement by Si is directly affected by the ROS-mediated process.

Si application enhances the resistance and tolerance of plants under drought stress by increasing plant defense responses, such as those of the antioxidant system, thereby reducing drought-induced oxidative stress^70,111^. In particular, Si increased the activities of SOD, CAT, and APX in wheat^29^, tomato^41^, chickpea^70^, rapeseed, and sunflower^34^, which in turn induced H_2_O_2_ production and lipid peroxidation underwater-deficient conditions. However, Si application decreased CAT, POD, and SOD activities and electrolyte leakage in soybean plants under drought stress^35^, indicating that oxidative damage induced by drought was alleviated by Si. Nevertheless, in drought-stressed wheat leaves, Si addition increased SOD activity while decreasing H_2_O_2_ and malondialdehyde (MDA) levels and electrolyte leakage^29,134,135^, suggesting that the different responses of enzyme activities to drought stress might be attributed to differences in plant species, growth stage, and stress degree. An essential role in alleviating oxidative damage in plants is also played by nonenzymatic antioxidants, and Si application increased GSH and AsA contents in drought-stressed wheat^29,134^. Moreover, activities of nonenzymatic antioxidants (e.g., AsA) in chickpea were induced by Si under drought stress conditions^70^, indicating that oxidative damage induced by drought was mitigated by Si by enhancing the activity of antioxidative systems. AsA reacts nonenzymatically with superoxide, H_2_O_2_, and singlet oxygen and reacts indirectly by regenerating tocopherols or synthesizing zeaxanthin in the xanthophyll cycle, which influences several enzyme activities and reduces the damage caused by the oxidative process through synergistic functions with those of other antioxidants^136^. The mechanisms by which Si activates antioxidant systems under drought stress are largely unknown; but it has been suggested that Si is involved in regulating the expression of genes related to the production and activation of antioxidant enzymes, such as *TaSOD*, *TaCAT*, and *TaAPX*^137^ under stress conditions. Moreover, exogenous application of Si alleviates drought stress through transcriptional regulation of enzymes involved in the ascorbate-glutathione (ASC–GSH) cycle (e.g., GS, GR, MDHAR, and DHAR) and in flavonoid secondary metabolism (e.g., PAL, CHS, F3H, DFR, and ANS)^137^.

To date, it has been found that Si can alleviate oxidative damage under drought stress by modulating plant antioxidant defense systems based on enzymatic or nonenzymatic constituents, which contributes to increased plant growth and whole-plant water balance. However, the importance of Si-mediated antioxidant defense largely depends upon plant species, cultivar, and growth stage, as well as the degree of stress and growth conditions. The underlying mechanisms by which Si alleviates oxidative damage under drought still need to be investigated, especially the role of Si in regulating the balance between ROS accumulation and antioxidant production.

## Conclusion and implications

**Fig. 4:**
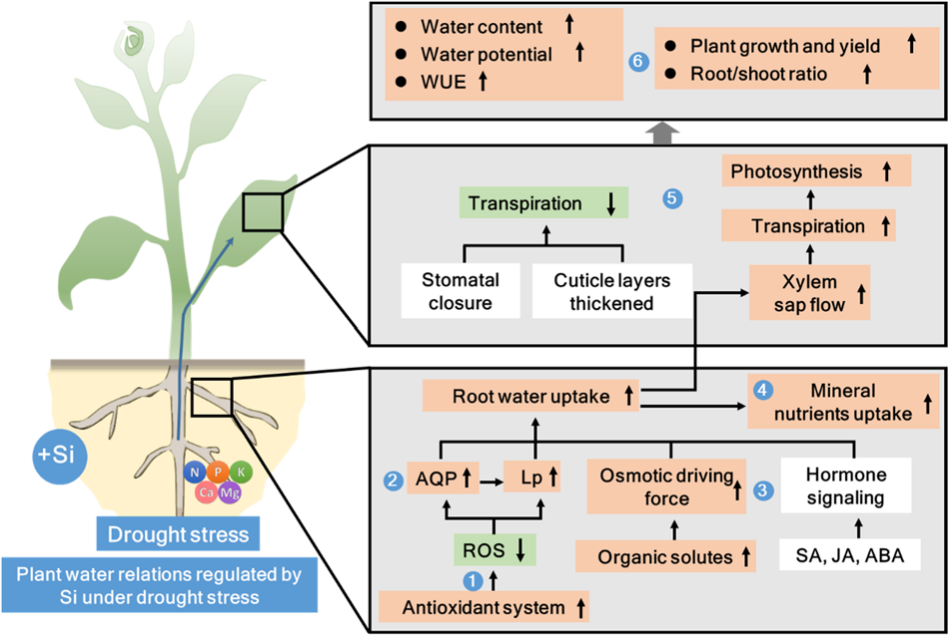
Key mechanisms involved in Si-triggered drought stress in plants. Plant water relations regulated by Si under drought stress conditions include (1) activation of antioxidant systems, (2) stimulation of gene expression and defense responses, (3) adjustment of osmotic processes and maintenance of homeostasis, (4) increases in nutrient uptake and maintenance of mineral balance, (5) regulation of photosynthesis and gas exchange, and (6) improvements in plant growth and water uptake

Drought stress is one of the major environmental factors that limits plant growth and crop productivity; this review summarizes the effects of Si on plant resistance and tolerance to drought stress (Table 1). Si application alleviates plant drought stress by (i) enhancing root water uptake, mainly through improving root growth, osmotic driving forces, hydraulic conductance, and mineral nutrient uptake, as well as by regulating aquaporin (AQP) activity and gene expression, (ii) regulating leaf transpirational water loss depending on root water status, and (iii) inducing plant defense responses through modification of signaling pathways and activation of antioxidant systems (Fig. 4). This makes Si application an attractive approach to improving plant water status and maintaining plant water balance under drought stress conditions. Understanding the interactions between Si application and plant responses will contribute to more efficient fertilization practices or enhanced stress tolerance of crop plants.

**Table 1:** Morphoanatomical, physiological, biochemical, and molecular processes involved in Si alleviation of drought stress in plants

Process	Resistance mechanism	Plant species	Response	Reference(s)
***Morphoanatomical***	**Stimulating seed germination**	Tomato (*Solanum lycopersicum* L.)	(+) 22~39%	[41]
		Wheat (*Triticum aestivum* L.)	(+) 13~37%	[44]
		Maize (*Zea mays* L.)	(NS)	[45]
		Lentil (*Lens culinaris* Medik.)	(+) 16~55%	[46]
		Rice (*Oryza sativa*)	(+) 8~10%	[47]
	**Improving root traits**	Upland rice (*Oryza sativa*)	Root dry weight (+) 23%	[28]
		Sunflower (*Helianthus annuus* L.)	Root dry weight (NS)	[34]
		Soybean (*Glycine max* L.)	Root dry weight (+) 34%	[35]
		Canola (*Brassica napus* L. cv. Okapi)	Root dry weight (+) 47%	[38]
		Rice (*Oryza sativa*)	Total root length (+) 40~65% Root surface area (+) 19~38% Root volume (+) 22~40%	[48]
		Sorghum (*Sorghum bicolor* L.)	Root dry weight (+) 74% ^[53]^ (+) 93% ^[59]^ (+) 110% ^[66]^ Root diameter (+) 16% ^[66]^	[53, 59, 66]
		Chickpea (*Cicer arietinum* L.)	Root dry weight (NS)	[70]
		Cucumber (*Cucumis sativus* L.)	Root surface area (+) 39% Root mean diameter (+) 18%	[73]
		Wheat (*Triticum aestivum* L.)	Root dry weight (NS)	[109]
	**Increasing shoot growth**	Upland rice (*Oryza sativa*)	Shoot dry weight (+) 18%	[28]
		Tomato (*Solanum lycopersicum* L.)	Shoot dry weight (+) 42%	[32]
		Soybean (*Glycine max* L.)	Shoot dry weight (+) 26%	[35]
		Canola (*Brassica napus* L. cv. Okapi)	Shoot dry weight (+) 76%	[38]
		Rice (*Oryza sativa*)	Shoot weight (+) 97-103% ^[48]^ Plant height (+) 4~9% ^[50]^	[48, 50]
		Sorghum (*Sorghum bicolor* L.)	Shoot dry weight (+) 41% ^[53]^ (+) 71% ^[59]^ (+) 78% ^[66]^	[53, 59, 66]
		Cucumber (*Cucumis sativus* L.)	Shoot dry weight (+) 32%	[68]
		Wheat (*Triticum aestivum* L.)	Plant height (NS)	[109]
	**Increasing the root/shoot ratio**	Upland rice (*Oryza sativa*)	(+) 9%	[28]
		Tomato (*Solanum lycopersicum* L.)	(NS)	[32]
		Soybean (*Glycine max* L.)	(+) 7%	[35]
		Sorghum (*Sorghum bicolor* L.)	(+) 4% ^[53]^ (NS) ^[59,66]^	[53, 59, 66]
***Physiological***	**Enhancing osmotic adjustment**	Upland rice (*Oryza sativa*)	Root osmotic adjustment (+) 134% Leaf osmotic adjustment (+) 63%	[28]
		Sorghum (*Sorghum bicolor* L.)	Root xylem osmotic adjustment (NS) ^[31]^ Leaf osmotic adjustment (+) 15% Root osmotic adjustment (+) 7% ^[53]^	[31, 53]
		Tomato (*Solanum lycopersicum* L.)	Root osmotic adjustment (+) 15%	[32]
		Cucumber (*Cucumis sativus* L.)	Root xylem osmotic adjustment (+) 39%	[73]
	**Enhancing water-use efficiency (WUE)**	Upland rice (*Oryza sativa*)	(+) 176%	[28]
		Canola (*Brassica napus* L. cv. Okapi)	(+) 20%	[38]
		Rice (*Oryza sativa*)	(+) 119%	[48]
		Maize (*Zea mays* L.)	(+) 30%	[93]
		Alfalfa (*Medicago sativa* L.)	(+) 20~36%	[105]
		Sorghum (*Sorghum bicolor* L.)	(NS)	[110]
	**Increasing the photosynthetic rate**	Upland rice (*Oryza sativa*)	(+) 260%	[28]
		Wheat (*Triticum aestivum* L.)	(+) 59%	[29]
		Tomato (*Solanum lycopersicum* L.)	(+) 143%	[32]
		Canola (*Brassica napus* L. cv. Okapi)	(+) 61%	[38]
		Rice (*Oryza sativa*)	(+) 37%	[48]
		Sorghum (*Sorghum bicolor* L.)	(+) 17% ^[91]^ (+) 118% ^[110]^	[91, 110]
	**Increasing water potential**	Upland rice (*Oryza sativa*)	(+) 17~27% ^[28]^	[28]
		Sorghum (*Sorghum bicolor* L.)	(+) 13% ^[31]^ (+) 16% ^[110]^	[31, 110]
		Rice (*Oryza sativa*)	(+) 15%	[48]
		Wheat (*Triticum aestivum* L.)	(+) 15% ^[29]^ (+) 40% ^[109]^	[29, 109]
	**Increasing hydraulic conductance**	Tomato (*Solanum lycopersicum* L.)	Root hydraulic conductance (+) 375%	[32]
		Cucumber (*Cucumis sativus* L.)	Root hydraulic conductance (+) 160%	[73]
		Sorghum (*Sorghum bicolor* L.)	Whole-plant hydraulic conductance (+) 52% ^[31]^ Root hydraulic conductance (+) 19% ^[91]^	[31, 91]
	**Modifying transpiration**	Upland rice (*Oryza* sativa)	(+) 32%	[28]
		Tomato (*Solanum lycopersicum* L.)	(+) 55%	[32]
		Soybean (*Glycine max* L.)	(+) 29%	[35]
		Rice (*Oryza sativa*)	(+) 19%	[48]
		Sorghum (*Sorghum bicolor* L.)	(+) 24% ^[31]^ (+) 25% ^[91]^	[31, 91]
		Alfalfa (*Medicago sativa* L.)	(+) 25-52%	[105]
		Maize (*Zea mays* L.)	(−) 30% ^[93]^ (−) 33~35% ^[102]^	[93, 102]
***Biochemical***	**Activating antioxidant systems**	Wheat (*Triticum aestivum* L.)	Root SOD (+) 22% CAT (+) 9%	[29]
		Tomato (*Solanum lycopersicum* L.)	Root SOD (+) 74% CAT (+) 65%	[32]
		Sunflower (*Helianthus annuus* L.)	Shoots CAT (+) 20%	[34]
		Canola (*Brassica napus* L. cv. Okapi)	Leaf SOD (+) 116% POD (+) 175% Root SOD (+) 20% POD (+) 27%	[38]
		Cucumber (*Cucumis sativus* L.)	Leaf GPX (+) 54% SOD (+) 21%	[69]
		Chickpea (*Cicer arietinum* L.)	Shoot SOD (NS) CAT (+) 106%	[70]
		Sorghum (*Sorghum bicolor* L.)	Root SOD (+)20% CAT (+) 27% APX (NS)	[91]
	**Activating nonenzymatic antioxidants**	Tomato (*Solanum lycopersicum* L.)	Root AsA (+) 62% GSH (+) 44%	[32]
		Sunflower (*Helianthus annuus* L.)	Shoot (+) 19%	[34]
		Chickpea (*Cicer arietinum* L.)	Shoot (+) 18%	[70]
	**Alleviating oxidative stress**	Wheat (*Triticum aestivum* L.)	Root H_2_O_2_ (−) 30%	[29]
		Tomato (*Solanum lycopersicum* L.)	Root H_2_O_2_ (−) 36~39% MDA (−) 16~45% O_2_^• ^^−^(−) 15%~23%	[32]
		Sunflower (*Helianthus annuus* L.)	Shoot H_2_O_2_ (−) 25% MDA (−) 11%	[34]
		Canola (*Brassica napus* L. cv. Okapi)	Leaf H_2_O_2_ (−) 9% MDA (−) 39% Root H_2_O_2_ (−) 47% MDA (−) 57%	[38]
		Cucumber (*Cucumis sativus* L.)	Leaf H_2_O_2_ (−) 18% ^[69]^~19% ^[68]^ MDA (−) 24% ^[69]^~52% ^[68]^ Root H_2_O_2_ (−)23% MDA (−) 22%	[68, 69]
		Chickpea (*Cicer arietinum* L.)	Shoot H_2_O_2_ (−) 42% MDA (−) 11% LOX (−) 8%	[70]
		Sorghum (*Sorghum bicolor* L.)	Root H_2_O_2_ (−) 50%	[91]
***Molecular***	**Regulating aquaporins**	Tomato (*Solanum lycopersicum* L.)	*SbPIP* relative expression (+) 60~165%	[32]
		Cucumber (*Cucumis sativus* L.)	*CsPIP* relative expression (+) 90~160%	[73]
		Sorghum (*Sorghum bicolor* L.)	*SbPIP* relative expression (+) 18~237% ^[31]^ *SbPIP* expression upregulated ^[91]^	[31, 91]
	**Modifying signaling pathways**	Sorghum (*Sorghum bicolor* L.)	Leaf PAs (+) 80% Root PAs (+) 67%	[53]
		Soybean (*Glycine max* L.)	Gibberellins (GAs) (+) 53% JA (−) 38% SA (−) 29%	[122]
		Barley (*Hordeum vulgare* cv.)	ABA ( +) 97% Phaseic acid (+) 74% Dehydro-phaseic acid (DPA) (+) 57% Cytokinin Ip (+) 76%	[124]
		Wheat (*Triticum aestivum* L.)	*TaSOD* relative expression (+) 26% *TaAPX* relative expression (+) 112% *TaCAT* relative expression (+) 200%	[137]

Positive (+), negative (−), and no effect (*NS* no significant difference) of silicon (Si) on plant drought resistance. The response data were calculated as follows: (Si supply—without Si supply)/without Si supply×100% (under drought stress)

Based on the current knowledge, the distribution of Si and its functions under stress conditions need further investigation, especially the differences among Si accumulators, intermediates, and excluders and the strategies for alleviating drought stress. In addition, published works are inconsistent, which may reflect the absence of a “one-size-fits-all” model for Si effects, with differences in mechanisms depending on species, genotypes, and the environment. This needs to be recognized before Si can be successfully applied to agriculture. Therefore, a systematic assessment of Si effects is needed, in which the effects could be linked to, for example, specific quantitative trait loci (QTLs) and/or transcriptomic assessments. In addition, to overcome global environmental changes and improve crop production, the application method of Si (e.g., soil-based or foliar) and its effect on plant tolerance and/or resistance under field conditions still need to be extensively investigated.
